# An epidemiological risk assessment of imported malaria cases and potential local transmission in Qatar

**DOI:** 10.1093/eurpub/ckae127

**Published:** 2025-01-13

**Authors:** Devendra Bansal, Nada Assaad, Hend Omar Mohamed, Muralitharan Shanmugakonar, Dorothy Pacate, Khider Mohamed, Perumal Balakrishnan, Redentor Cuizon Ramiscal, Nandakumar Ganesan, Maha Hammam M A Al-Shamali, Ali A Sultan, Waqar Munir, Mohammed Abukhattab, Francis Schaffner, Muna A Al-Maslamani, Hamad Eid Al-Romaihi, Mohammed Al-Thani, Fatima Al Khayat, Elmoubashar Abd Farag

**Affiliations:** Health Protection and Communicable Diseases Control Department, Ministry of Public Health, Doha, Qatar; Department of Biological and Environmental Sciences, Qatar University, Doha, Qatar; Weill Cornell Medicine-Qatar, Doha, Qatar; Laboratory Animal Research Center, Qatar University, Doha, Qatar; Health Protection and Communicable Diseases Control Department, Ministry of Public Health, Doha, Qatar; Health Protection and Communicable Diseases Control Department, Ministry of Public Health, Doha, Qatar; Department of Biological and Environmental Sciences, Qatar University, Doha, Qatar; Health Protection and Communicable Diseases Control Department, Ministry of Public Health, Doha, Qatar; Health Protection and Communicable Diseases Control Department, Ministry of Public Health, Doha, Qatar; Health Protection and Communicable Diseases Control Department, Ministry of Public Health, Doha, Qatar; Weill Cornell Medicine-Qatar, Doha, Qatar; Infectious Disease Section, Communicable Disease Center, Hamad Medical Corporation, Doha, Qatar; Infectious Disease Section, Communicable Disease Center, Hamad Medical Corporation, Doha, Qatar; Francis Schaffner Consultancy, Riehen, Switzerland; Infectious Disease Section, Communicable Disease Center, Hamad Medical Corporation, Doha, Qatar; Health Protection and Communicable Diseases Control Department, Ministry of Public Health, Doha, Qatar; Health Protection and Communicable Diseases Control Department, Ministry of Public Health, Doha, Qatar; Department of Biological and Environmental Sciences, Qatar University, Doha, Qatar; Health Protection and Communicable Diseases Control Department, Ministry of Public Health, Doha, Qatar

## Abstract

Preventing local transmission of malaria from imported cases is crucial for achieving and maintaining malaria elimination. This study aimed to investigate the epidemiological characteristics of imported malaria cases and assess the distribution of malaria vectors in Qatar. Data from January 2016 to December 2022 on imported malaria, including demographic and epidemiological characteristics, travel-related information, and diagnostic results, were collected and analysed using descriptive statistics. Field surveys conducted in 2021–22 collected mosquitoes using various traps across Qatar. The collected samples underwent morphological and molecular characterization at Qatar University. A total of 2693 cases were reported, with a mean incidence of 13.5/100 000 population, decreasing from 18.8/100 000 in 2016 to 5.5/100 000 in 2020. Most cases were *Plasmodium vivax* (57.4%) followed by *P. falciparum* (40.4%). The median age was 32.9 ± 12.5 years, primarily males (86.7%), expatriates (99.6%) and notified during the hot months (July to September). Cases were mainly imported from the Eastern Mediterranean Region followed by the African and South-East Asia Region with no deaths and indigenous cases. *Anopheles stephensi* was identified as a widely distributed species, but none carried the *Plasmodium* pathogen. Despite no reports of local transmission, the presence of *An. stephensi* and favourable environmental conditions pose a risk in Qatar. Strengthening surveillance for imported malaria and reviewing epidemic protocols are necessary. Conventional field studies are imperative to address knowledge gaps in *Anopheles* mosquito ecology and biting habits in Qatar, accurately assessing the risk of local malaria transmission to support Qatar’s malaria-free status.

Key points

- Persistent risk factors, such as imported cases, the presence of the malaria vector, climate change, *P. vivax* relapses, high genetic diversity, and drug resistance, pose a threat in Qatar.
- It is necessary to prioritize vector control, monitoring, and surveillance to prevent the reintroduction of malaria transmission.
- There is a need for an active case and vector surveillance program, especially considering the significant expatriate population.

## Introduction

Malaria remains among the most important global public health concerns in tropical and subtropical climates. The World Health Organization estimates 249 million cases in 2022 compared to 244 million in 2021 and 608 000 deaths in 2022 compared to 619 000 in 2021 attributed to malaria. The notable upsurge in malaria incidence can be linked to the disruptions in the delivery of malaria control interventions due to the coronavirus disease 2019 (COVID-19) pandemic.^1^

African region account for most of the cases and deaths linked to malaria, followed by South and South-East Asia region.^1^ In the Gulf Cooperation Council (GCC) member states, namely Qatar, Saudi Arabia, Bahrain, Kuwait, Oman, and the United Arab Emirates (UAE), imported cases of malaria have also been reported. These cases primarily come from countries in South/South-East Asia and Africa.^2^^,^^3^ However, only Southwestern Saudi Arabia has documented the occurrence of both imported and local malaria cases.^2^^,^^4^ In addition, potential malaria vectors have also been detected in Bahrain, UAE, Saudi Arabia, and Qatar.^^5–8^^

In the past few decades, the GCC countries have experienced a massive influx of foreign workers as the countries underwent tremendous economic growth and development. Their arid climate, booming commercial and tourism sector, and high level of urbanization are among the many similarities shared between these nations.^9^^,^^10^ The existing and constantly evolving dynamics in the GCC create favourable conditions for the survival of vectors, potentially accelerating disease transmission, making vector-borne diseases (VBDs), including malaria, of public health importance to the region.^2^

Climate change can potentially affect the spatial distribution of vectors, which can impact the spread of VBDs, such as malaria.^2^^,^^11^ The continuously changing ecological and climatic conditions can also increase the risk of vector (re-)introduction and survival globally.^12^^,^^13^ The adverse effects of climate change can be further exacerbated by anthropogenic factors such as rapid urbanization, land development, and population expansion. In addition, a suitable environment along with imported malaria cases may also cause the recurrence of malaria in non-endemic countries like Qatar.

Although Qatar has been malaria-free since the 1970s, the risk of reintroduction exists due to malaria receptivity.^5^^,^^14^^,^^15^ Moreover, the expatriate population in Qatar has been steadily increasing over the past decade and continues to grow due to the rapid development in the country. Consequently, strict surveillance measures are necessary. The Ministry of Public Health (MoPH) in Qatar conducts an active epidemiological investigation of all VBD cases using a structured epidemiological and environmental investigation form that includes information on mosquito collection, clinical history, and travel history to classify the case (local or imported) and identify factors that may be associated with transmission risk, and which can be acted upon. This retrospective investigation aims to gain an understanding of the local transmission dynamics of malaria, the intensity of the environmental factors involved, and the necessary control measures that need to be implemented in the country to prevent the spread of malaria.

## Methods

### Health facilities and malaria data collection

The state of Qatar is a peninsular country with a population of 3.08 million, of which 88% are expatriates.^16^ This region has a desert climate, characterized by long and dry summers and mild winters. The average annual rainfall ranges between 72 and 100 mm, mainly in winter and spring (December to April). The average temperature throughout the year is 29°C. This study is a retrospective review of malaria cases diagnosed between January 2016 and December 2022 at various healthcare institutions in Qatar. These include the Hamad Medical Corporation (HMC), which manages 14 specialized hospitals: The Primary Health Care Corporation with 28 health centres, and other government, semi-government, and private health institutions. All malaria cases diagnosed were immediately reported using an electronic surveillance system to the Health Protection and Communicable Diseases Control Department at the MoPH. The data were analysed to describe the epidemiological features, incorporating demographic characteristics such as age, gender, nationality (either Qatari, or expatriates residing in Qatar for at least 1 year), travel history, time of malaria reporting, and *Plasmodium* species. Ethical approvals were obtained from the MoPH Institutional Review Board (ERC-930-3-2020 and ERC-829-3-2022) and Qatar University Institutional Review Board (QU-IRB 1421-E/20).

### Laboratory procedure and case management of malaria

The blood samples were examined for the presence of the malaria parasite using microscopic examination, with Giemsa staining of thin and/or thick blood films, as well as a Rapid Diagnostic Test. All patients diagnosed with malaria were treated with antimalarial medication according to the current guidelines of HMC, as previously published.^3^

### Mosquito collection and morphological identifications

Mosquito traps were set up at 58 sampling stations in Qatar, including agricultural farms, animal farms, plant nurseries, and residential and workplace areas of malaria cases. These stations were located in all eight municipalities in Qatar. Mosquitoes were collected using CDC traps (employing CO_2_ dry ice canisters) (BioQuip Products, Inc., Rancho Dominguez, CA) and Blackhole traps (applying UV light) (Bio-Trap Inc., Seoul, Korea) between 2021 and 2022. All traps were placed at the designated collection sites before sunset and retrieved the following day before sunrise. The samples were then transferred directly to the Entomology lab at Qatar University for further morphological identification. This identification process involved using a Leica M125 stereomicroscope, a cold chain system, and taxonomical keys.^^17–20^^

### Characterization of *Anopheles* spp. and detection of malaria parasite by molecular assays

All morphologically identified *Anopheles* female mosquitoes were further confirmed by molecular assay and infection by *Plasmodium* parasite was investigated.

#### DNA preparation of mosquito samples

DNA extraction was performed from the head and thorax of the mosquito samples using the Nucleospin DNA insect commercial kit (Macherey-Nagel, Germany), following the manufacturer’s instructions. The extracted DNA was stored at −20°C for further use.

#### Molecular characterization of *Anopheles* mosquitoes

To confirm the presence of *Anopheles* spp., the mitochondrial cytochrome C oxidase subunit 1 gene (COI) was amplified using specific primers (Supplementary Table S1). The conventional polymerase chain reaction (PCR) was conducted with a 20 µl reaction mixture comprising 10 picomoles of forward and reverse COI-specific primers, 100 ng of extracted DNA, 1 µl of Taq DNA polymerase and 1× gold PCR master mix (Applied Biosystems, USA). PCR amplification was initiated at 94°C for 3 min, followed by 30 cycles (denaturation at 94°C for 40 s, annealing at 48°C for 30 s, and extension at 72°C for 40 s), with a final extension for 4 min at 72°C. Positive and negative controls were included in the amplification reaction. PCR Purification and standard Sanger sequencing were performed at Macrogen (Seoul, South Korea). The generated sequence data were subjected to a similarity search using the Basic Local Alignment Search Tool (BLAST) and was compared to reference sequences of *Anopheles* spp. from nearby countries retrieved from Genbank.^21^ The maximum likelihood tree was constructed using Mega X software.^22^

#### Detection of malaria parasite

Genus and species-specific nested PCR was performed to detect malaria parasite species (*Plasmodium falciparum, P. vivax, P. ovale*, and *P. malariae*) in *Anopheles* mosquitoes. The details of the nested PCR primers and annealing conditions can be found in Supplementary Table S1. In the primary PCR, a volume of 20 µl was used, containing 10 picomole of each primer (rPLU1 and rPLU5), 5 µl of DNA template, 1× gold PCR master mix, and 1 µl of Taq DNA polymerase (Applied Biosystems, USA). The primary product (2 µl) was then used for nested PCR using the primers (rPLU3 and rPLU4) and species-specific primers (rFAL1, rFAL2, rVIV1, rVIV2, rMAL1, rMAL2, rOVA1, and rOVA2). The nested PCR products were visualized under a UV transilluminator after resolving them on a 1.5% agarose gel. A negative control of nuclease-free water was included in each PCR reaction.

Real-time PCR-based *Plasmodium* qPCR commercial kits (Genesig, UK) were also used to identify *Plasmodium* species, according to manufacturers’ instructions. The kit targets the 18S ribosomal gene, enabling the detection of various *Plasmodium* species. The reaction was performed in 20 µl volume, and the mixture consisted of *Plasmodium*-specific Primer/Probe mix, 1× precision plus qPCR master mix, and 50 ηg of DNA sample. Additionally, a positive and internal control DNA was included in the qPCR reaction at different dilutions, ranging from 2 to 2×10^6^ copy numbers per microliter. Samples of the same species were pooled and assessed in qPCR analysis for *Plasmodium* species identification. The following thermal cycle conditions were employed: enzyme activation at 95°C for 2 min followed by 50 cycles of 95°C for 10 s and 60°C for 60 s. Fluorogenic data was collected through FAM and VIC channels.

### Statistical analysis

All the collected data were entered into Microsoft Excel, and descriptive statistical analysis was performed for all important and relevant variables using IBM SPSS version 22 (SPSS Inc. Chicago, IL). The data were reported as means ± standard deviation and described as numbers and percentages.

## Results

### Characteristics of demographic and epidemiological distribution

A total of 2693 malaria cases were reported between January 2016 and December 2022. These cases were analysed to determine their epidemiological characteristics, as shown in Table 1. The primary causative organism identified was *P. vivax*, accounting for 57.4% of reported cases. This was followed by *P. falciparum* (40.4%), mixed infections of *P. vivax* and *P. falciparum* (1.5%), *P. ovale* (0.3%), *P. malariae* (0.2%), and mixed infections of *P. vivax* and *P. ovale* (0.1%) (Table 1).

**Table 1.** Demographic profile of imported malaria cases in the State of Qatar between 2016 and 2022 [total number (*n*) of cases = 2693]

Variables	*n* (%)
**Gender**	
Male	2335 (86.7)
Female	358 (13.3)
**Nationality**	
Qatari	10 (0.4)
Expatriates	2683 (99.6)
**Age (years)**	
<15	122 (4.5)
15–29	1049 (39.0)
30–44	1040 (38.6)
>44	482 (17.9)
**Malaria species**	
*P. vivax*	1547 (57.4)
*P. falciparum*	1088 (40.4)
*P. malariae*	6 (0.2)
*P. ovale*	8 (0.3)
Mixed (PV+PF)	41 (1.5)
Mixed (PV+PO)	3 (0.1)
**Cases by season**	
January to March	423 (15.7)
April to June	619 (23.0)
July to September	985 (36.6)
October to December	666 (24.7)

**Figure 1.**
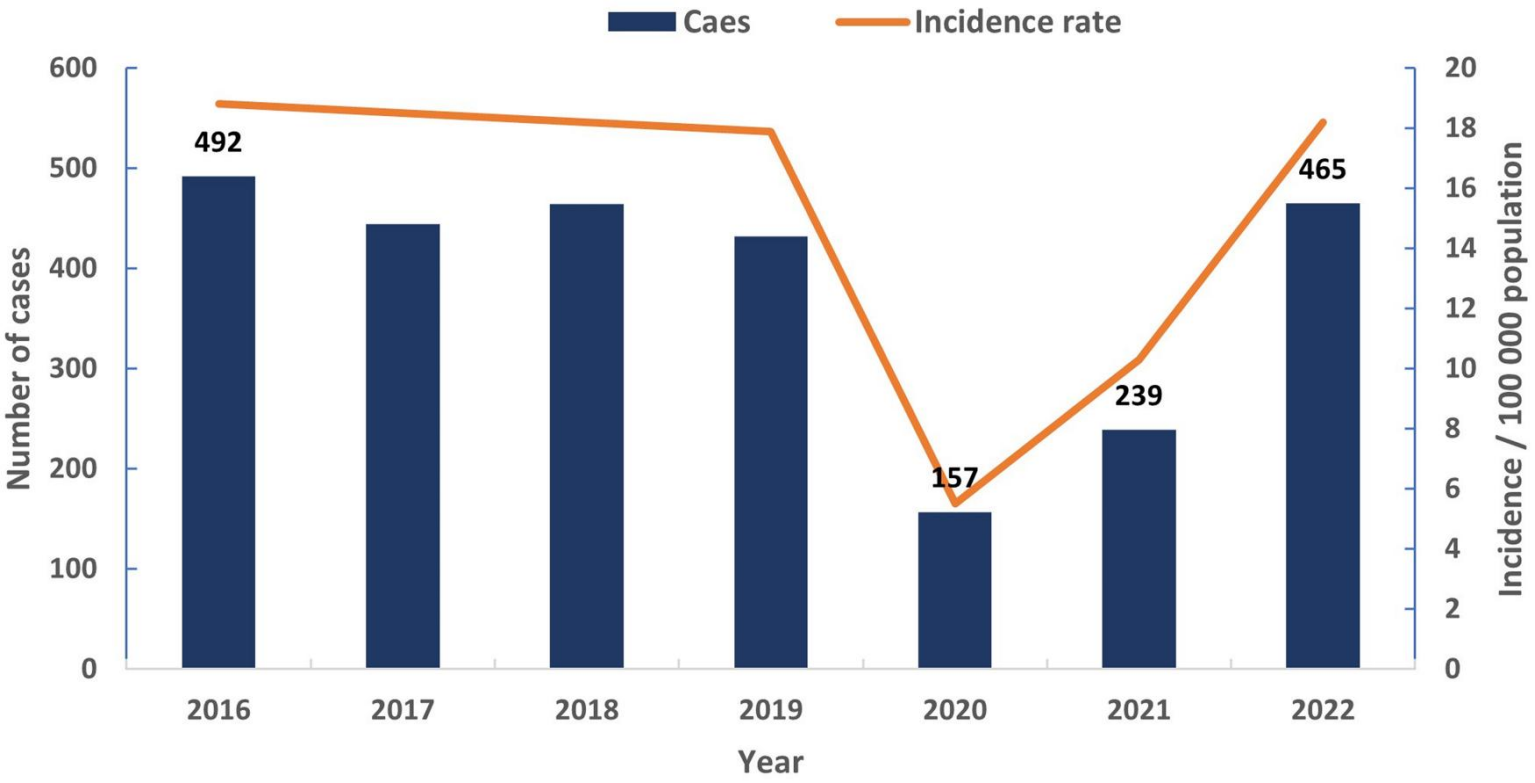
Annual distribution of reported cases of imported malaria and incidence rate per 100 000 population in the State of Qatar, 2016–22 (*N* = 2693).

It was found that all the reported malaria cases in the country were imported, and no malaria-associated deaths were reported. The mean incidence was estimated to be 13.5/per 100 000 population. A declining trend that culminates with the lowest number of 157 cases recorded in the year 2020 (incidence rates decreased from 18.8/100 000 in 2016–5.5/100 000 in 2020) was observed, followed by an increase (239 cases) in 2021 and 2022 (465 cases; Fig. 1).

The demographic features of malaria indicated that most reported cases were males (86.9%), reflecting the high number of male workers, including both blue- and white-collar workers, who form much of the immigrant population in Qatar. Most of the cases were aged between 15–44 years (77.6%) and the incidence rate among this age group was 95.6/100 000 population.

Furthermore, season-wise, more than a third (36.6%) of the annual cases were reported between July and September (Table 1). Among the nationalities, most cases were expatriates (99.6%) particularly from the Eastern Mediterranean Region (44.8%), followed by the South-East Asia Region (27.9%) and the Africa Region (25.9%) (Supplementary Fig. S1).

### Morphological and molecular characterization of vector species

**Figure 2.**
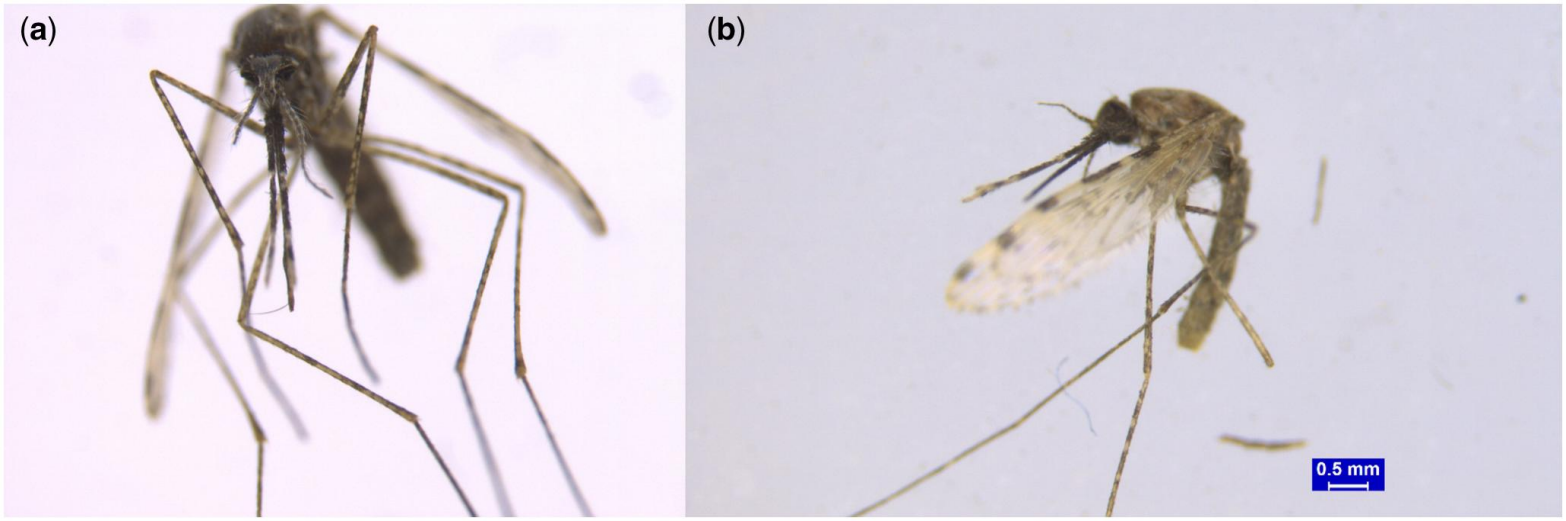
*Anopheles stephensi* female under the stereomicroscope. (a) Ventral view and (b) lateral view.

**Figure 3.**
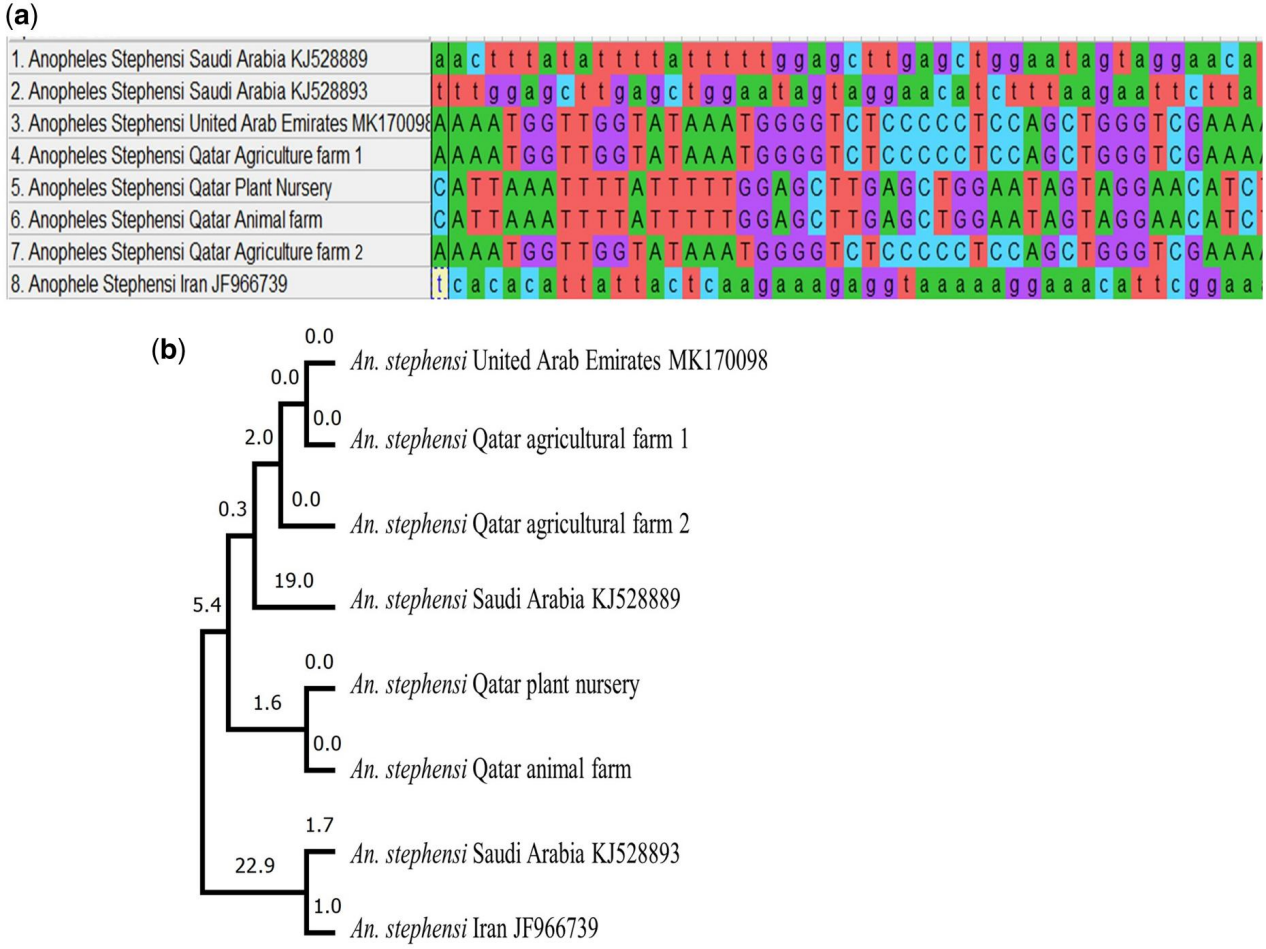
Analysis of COI sequences of *Anopheles stephensi*. (a) Sequences alignment of specimens collected from different areas in Qatar and nearby countries. (b) Evolutionary relationship of sequences from different areas in Qatar and nearby countries.

A total of 1947 mosquitoes were collected during the study period, and the results revealed that the *Culex* species were the most common, followed by *Anopheles* and *Aedes*. Moreover, *Anopheles* spp. were identified as *Anopheles stephensi,* a malaria-specific mosquito vector, by morphological analysis (Fig. 2) and confirmed through molecular identification COI sequencing (Fig. 3). The generated COI sequences were compared with reference sequences from nearby countries obtained from the GenBank to construct a phylogenetic tree (Fig. 3). The analysis reveals that the samples collected from various regions in Qatar cluster together with the sequences from the United Arab Emirates and Saudi Arabia.

### Detection of malaria parasite by molecular assays

In total, 86 samples were subjected to nested PCR and qPCR analysis, and all were negative for *Plasmodium* species.

## Discussion

Malaria continues to be the most important and prevalent VBD in many developing countries. This study presents the epidemiological characteristics of imported malaria cases diagnosed in Qatar and determines the distribution of malaria vectors and pathogen detection.

A total of 2693 imported malaria cases were reported between January 2016 and December 2022. Most of these cases were found among male expatriates between the ages of 25 and 54, which reflects the country’s large population of single, male migrant blue-collar workers^16^ and corroborates with the cases reported between 2008 and 2015 in Qatar.^3^ Cases among Qatari nationals were very low (10, 0.4%), likely due to less travel to endemic countries; however, all of the confirmed cases had a travel history to endemic countries.

In the last two decades, the annual number of imported malaria cases in Qatar gradually increased from 216 cases in 2008 to 728 cases in 2013.^3^ However, the number of malaria cases has declined to 432 cases in 2019 (Fig. 1). Notably, the number of imported malaria cases in Qatar dropped from 432 in 2019 to 157 in 2020. This considerable decline may be due to travel restrictions and under-reporting of the disease as the COVID-19 pandemic took precedence, competing for public health resources worldwide. However, after COVID-19 cases began to decline, flight restrictions were lifted, and malaria cases again saw a significant increase, bringing the numbers to 239 in 2021 and 465 in 2022, as a direct result of resumed travel to and from endemic countries.

In terms of the seasonal distribution of malaria cases, a gradual increase was observed from July to September (36.6%). This rise in cases can be attributed to the return of expatriates to Qatar during these months after their summer vacation in their home countries. This trend remains consistent with previous reports of imported malaria cases in Qatar and other GCC countries.^2^^,^^3^^,^^6^ The increase in cases during the summer months poses a potential risk, as it has been observed that the higher temperatures and humidity during this season contribute to a higher abundance of both *Anopheles* and *Aedes* mosquitoes.^11^

It is well known that *P. falciparum* is the most prevalent malaria parasite in the African region, while *P. vivax* is dominant in South and South-East Asia countries.^1^ In the present study, *P. vivax* (57.4%) was the primary malaria parasite detected in Qatar, followed by *P. falciparum* (40.4%). In addition, *P. vivax* was diagnosed mostly among individuals from the Eastern Mediterranean Region and the South-East Asia Region. On the other hand, *P. falciparum* was more commonly detected among individuals from the African region, which is consistent with previous reports from Qatar and GCC.^3^^,^^^23–25^^

The study reported *Culex* mosquitoes as the most prevalent, followed by the *Anopheles* and *Aedes* types. These findings are consistent with recent entomological surveys conducted in Qatar.^5^^,^^11^^,^^14^^,^^15^ Although the existence of effective malaria vectors, *An. stephensi* and *An. multicolor* species were reported in some regions of Qatar, albeit in low density.^14^^,^^15^ However, in the present study, *An. stephensi* was found throughout the country, which raises the potential risk for local malaria transmission. Interestingly, none of the *An. stephensi* mosquitoes were found positive for *Plasmodium* pathogens.

In Qatar, the risk of local malaria transmission persists due to factors such as imported cases of malaria, mainly *P. vivax* from South-East Asia countries, the presence of malaria vectors, and climate change. The Chesson strain of *P. vivax*, which originates from South-East Asia, is known to result in more relapses due to its short latency period, poses a significant challenge to effectively controlling local malaria transmission.^26^ Imported cases of *P. falciparum* malaria in Qatar have also shown high genetic diversity, resistance to chloroquine and sulphadoxine-pyrimethamine, and the presence of early- and late-stage gametocytes, which is a threat to the re-establishment of drug-resistant malaria.^^27–30^^ This scenario highlights how mass international migration can re-introduce malaria to areas with limited or no local transmission.^31^ Furthermore, human mobility has been observed to influence disease exposure within a population, as it can potentially increase the spread of vectors and the pathogens they carry.^32^ Therefore, monitoring and routine surveillance of malaria cases and vectors in Qatar are crucial for safeguarding public health after the FIFA World Cup 2022. Tailoring interventions based on the epidemiological profile of malaria cases (including how, where, when, and which people) and its vectors would significantly impact the prediction and management of anticipated cases.^33^^,^^34^

The limitations of our study should be taken into account when interpreting the results. Firstly, as this is a retrospective study, we have not been able to conduct follow-ups with patients. Instead, we rely mainly on patient notification reports from the electronic surveillance system at MoPH. Secondly, it is important to note that since no asymptomatic infections have been reported to the MoPH surveillance system, caution should be exercised when interpreting incidence rate estimates.

In conclusion, this study presents the epidemiological investigation and risk assessment results that will further guide the active surveillance and control strategy of imported malaria in Qatar. Although no local malaria cases have yet been reported in Qatar, it is crucial to prioritize vector control, monitoring, and surveillance to prevent the reintroduction of malaria.^35^ Considering that a significant number of expatriates in Qatar come from malaria-endemic countries and the presence of *Anopheles* vector, it is necessary for the country to implement an active case and vector surveillance program to prevent and control outbreaks. Qatar's current healthcare system is well-equipped to address any potential threats to public health in its present state.

## Supplementary Material

ckae127_Supplementary_Data

## Data Availability

All data underlying the results are available as part of the article and no additional source data are required.
