# Supplementary material for: An epidemiological risk assessment of imported malaria cases and potential local transmission in Qatar
Source: Eur J Public Health. 2025 Jan 13;35(Suppl 1):i35–40. doi: 10.1093/eurpub/ckae127 (PMC11725959; doi:10.1093/eurpub/ckae127)
Supplement: ckae127_Supplementary_Data [file ckae127_supplementary_data.zip › ckae127_Supplementary_Data/ejph-2024-02--0078-File007.docx]

### Supplementary Table 1. Primer sequences for conventional and nested PCR analysis

| **Gene** | **Primer Sequence** | **Annealing Temperature** |
| --- | --- | --- |
| COI | *LCO1490F*: 5’GGTCAACAAATCATAAAGATATTGG 3’ | 48^0^C |
|  | *HCO2198R*: 5’ TAAACTTCAGGGTGACCAAAAAATCA ‘3 |  |
| *Plasmodium* genus-specific | *rPLU1* – 5’TCA AAG ATT AAG CCA TGC AAG TGA ‘3 | 55^0^C |
|  | *rPLU5* – 5’CCT GTT GTT GCC TTA AAC TCC ‘3 |  |
|  | *rPLU3* – 5’ TTT TTA TAA GGA TAA CTA CGG AAA AGC TGT ‘3 | 58^0^C |
|  | *rPLU4* – 5’TAC CCG TCA TAG CCA TGT TAG GCC AAT ACC ‘3 |  |
| *P. falciparum* | *rFAL1*- 5’ TTA AAC TGG TTT GGG AAA ACC AAA TAT ATT ‘3 | 62^0^C |
|  | *rFAL2* – 5’ACA CAA TGA ACT CAA TCA TGA CTA CCC GTC ‘3 |  |
| *P. vivax* | *rVIV1* – 5’CGC TTC TAG CTT AAT CCA CAT AAC TGA TAC ‘3 | 62^0^C |
|  | *rVIV2*- 5’ ACT TCC AAG CCG AAG CAA AGA AAG TCC TTA ‘3 |  |
| *P. malariae* | *rMAL1*– 5’ ATA ACA TAG TTG TAC GTT AAG AAT AAC CGC ‘3 | 58^0^C |
|  | *rMAL2* – 5’ AAA ATT CCC ATG CAT AAA AAA TTA TAC AAA ‘3 |  |
| *P. ovale* | *rOVA1* – 5’ ATC TCT TTT GCT ATT TTT TAG TAT TGG AGA ‘3 | 55^0^C |
|  | *rOVA2* – 5’ GGA AAA GGA CAC ATT AAT TGT ATC CTA GTG ‘3 |  |
