# Supplementary figures and images for: An epidemiological risk assessment of imported malaria cases and potential local transmission in Qatar
Source: Eur J Public Health. 2025 Jan 13;35(Suppl 1):i35–40. doi: 10.1093/eurpub/ckae127 (PMC11725959; doi:10.1093/eurpub/ckae127)

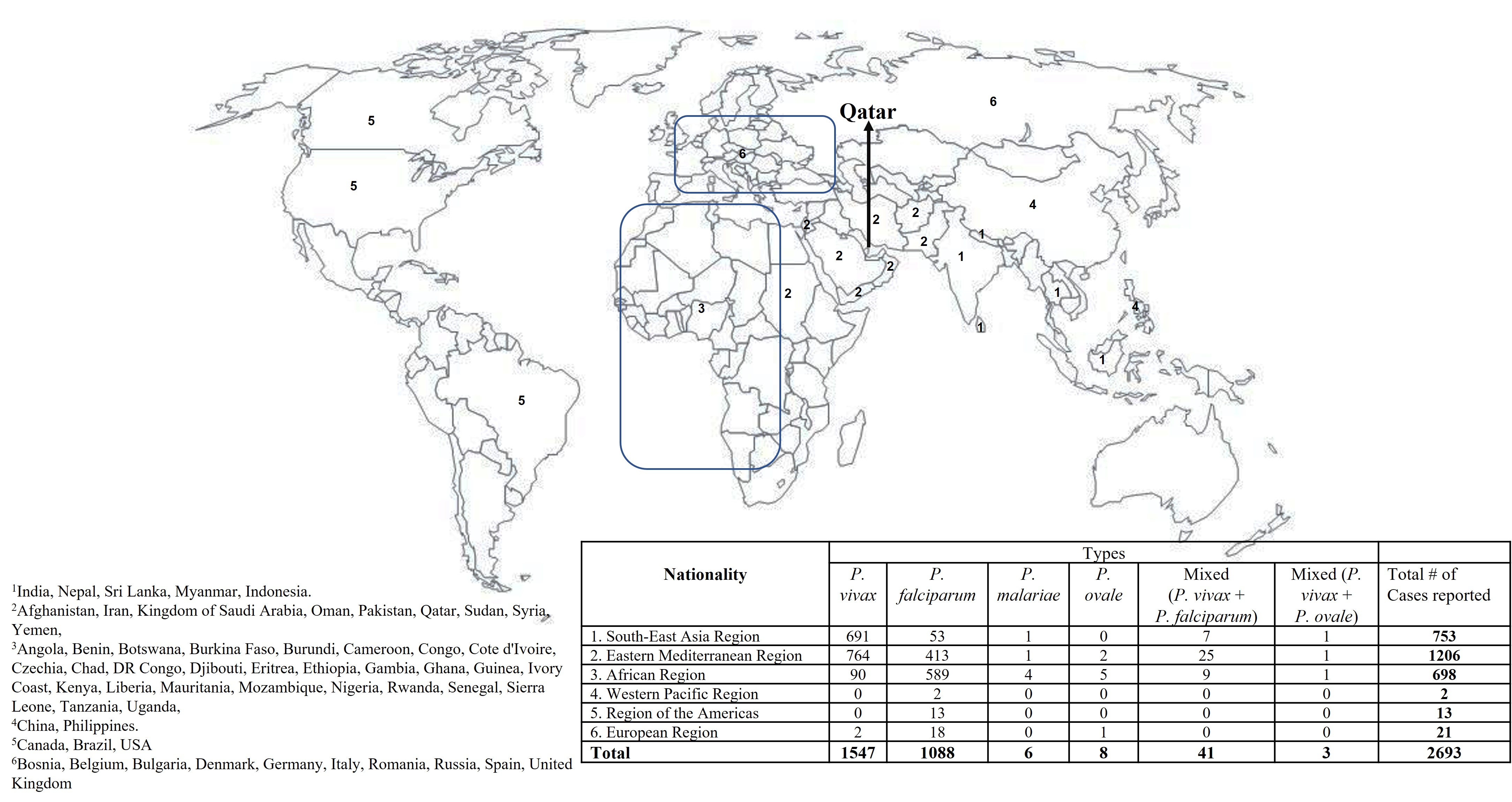

Supplement: ckae127_Supplementary_Data [file ckae127_supplementary_data.zip › ckae127_Supplementary_Data/ejph-2024-02--0078-File006.tif]
